# Tryptophan-Kynurenine Metabolism and Insulin Resistance in Hepatitis C Patients

**DOI:** 10.1155/2013/149247

**Published:** 2013-09-04

**Authors:** G. F. Oxenkrug, W. A. Turski, W. Zgrajka, J. V. Weinstock, P. Summergrad

**Affiliations:** ^1^Psychiatry and Inflammation Program, Department of Psychiatry, Tufts Medical Center, Tufts University, Boston, MA 02111, USA; ^2^Department of Experimental and Clinical Pharmacology, Medical University, 20-090 Lublin, Poland; ^3^Department of Toxicology, Institute of Rural Health, 20-090 Lublin, Poland; ^4^Division of Gastroenterology/Hepatology, Tufts Medical Center, Tufts University, Boston, MA 02111, USA

## Abstract

Chronic hepatitis C virus (HCV) infection is associated with 50% incidence of insulin resistance (IR) that is fourfold higher than that in non-HCV population. IR impairs the outcome of antiviral treatment. The molecular mechanisms of IR in HCV are not entirely clear. Experimental and clinical data suggested that hepatitis C virus per se is diabetogenic. However, presence of HCV alone does not affect IR. It was proposed that IR is mediated by proinflammatory cytokines, mainly by TNF-alpha. TNF-alpha potentiates interferon-gamma-induced transcriptional activation of indoleamine 2,3-dioxygenase, the rate-limiting enzyme of tryptophan- (TRP-) kynurenine (KYN) metabolism. Upregulation of TRP-KYN metabolism was reported in HCV patients. KYN and some of its derivatives affect insulin signaling pathways. We hypothesized that upregulation of TRP-KYN metabolism might contribute to the development of IR in HCV. To check this suggestion, we evaluated serum concentrations of TRP and KYN and HOMA-IR and HOMA-beta in 60 chronic HCV patients considered for the treatment with IFN-alpha. KYN and TRP concentrations correlated with HOMA-IR and HOMA-beta scores. Our data suggest the involvement of KYN and its metabolites in the development of IR in HCV patients. TRP-KYN metabolism might be a new target for prevention and treatment of IR in HCV patients.

## 1. Introduction

 Hepatitis C patients have fourfold higher incidence of insulin resistance (IR) than non-HCV population, that is, healthy controls or chronic hepatitis B patients [1]. IR is the major feature of the metabolic syndrome (diabetes type 2, obesity, hypertension, and cardiovascular disorders). HCV-associated IR may lead to resistance to antiviral therapy, hepatocarcinogenesis, and extrahepatic complications [2, 3].

The molecular mechanisms whereby HCV infection leads to IR are not entirely clear. Experimental and clinical findings indicated that hepatitis C virus per se is diabetogenic [4, 5]. However, presence of HCV alone does not affect IR [6]. It was suggested that increased production of proinflammatory cytokines, especially TNF-alpha, contributes to the development of IR in HCV patients [7]. TNF-alpha potentiates interferon-gamma- (IFNG-) triggered transcriptional induction of indoleamine 2,3-dioxygenase (IDO), the rate-limiting enzyme of tryptophan- (TRP-) kynurenine (KYN) metabolism [8]. Upregulated IDO expression in the dendritic cells [9] and in the liver [10] and increased serum KYN : TRP ratio (KTR) [10] were reported for HCV patients. Review of clinical and experimental data suggested that KYN and some of its derivatives affect biosynthesis, release, and activity of insulin [11]. We suggested that upregulated TRP-KYN metabolism might be one of the mechanisms of IR in HCV patients [12]. To check this suggestion, we evaluated serum TRP and KYN concentrations and IR and pancreatic beta-cell function in HCV patients.

## 2. Methods

 Participants were recruited from HCV patients considered for starting a treatment with pegylated interferon-alpha and ribavirin. The study was approved by Tufts Medical Center IRB, and written consents were obtained for participation in the study. Blood samples were collected after 12 hrs of fasting.

### 2.1. Assessment of IDO Activity

IDO activation results in decrease of TRP and increase of KYN and, therefore, in elevation of KTR that is used as a marker of IDO activity in clinical studies [13]. However, there are some peculiarities related to the use of KTR as a marker of IDO activity in HCV patients. Increased KTR was reported in HCV patients but without data on serum TRP and KYN levels [10]. On the other hand, the decreased concentrations of both TRP and KYN in serum and macrophages and, consequently, decreased KTR were observed in HCV [14]. In the largest, so far, study, concentrations of KYN in 176 patients were significantly higher those than in healthy controls, whereas the levels of TRP were comparable in the two groups. Authors suggested that in HCV patients serum KYN level can be used as a marker of IDO activity [9].

Serum TRP, KYN, and kynurenic acid (KYNA) concentrations were evaluated by HPLC-UV-fluorimetric method [15]. 

### 2.2. Assessment of IR

IR was assessed by homeostatic model assessment index, version 2 (HOMA2-IR), and pancreatic beta-cell function by HOMA-beta index, based on fasting glucose and insulin levels, using the computer-based solution of the model provided by the Diabetes Trials Unit, Oxford Center for Diabetes, Endocrinology, and Metabolism (http://www.dtu.ox.ac.uk/index.php?maindoc=/homa/history.php) [16]. Serum glucose was measured using an enzymatic, kinetic reaction on the Olympus AU400e with Olympus Glucose Reagents (OSCR6121) (Olympus America Inc., Melville, NY, USA). Serum insulin is measured using the Immulite 1000 Insulin Kit (LKIN1) on the Immulite 1000 (Siemens Medical Solutions Diagnostics, Los Angeles, CA, USA).

### 2.3. Statistical Treatment

Quantitative data are presented using median (50th percentile) and minimum-maximum range. Nonparametric tests (Wilcoxon and Mann-Whitney *U*) were used to assess correlations for nonnormally distributed data. 

## 3. Results

There were 42 male and 18 female American Caucasian HCV patients, 52.2 ± 7.45 years of age. Forty-eight patients had HCV genotype 1 or 4, and twelve patients had HCV genotype 2 or 3. None of the patients have been diagnosed with diabetes mellitus. 20 out of 60 patients had HOMA2-IR >2.

Serum KYN concentrations correlated with scores of HOMA2-IR and HOMA-beta (Table 1). TRP (KYN precursor) but not KYNA (immediate metabolite of KYN) [17, 18] correlated with HOMA2-IR and HOMA-beta. HOMA2-IR strongly correlated with HOMA-beta scores (Table 1). There was no correlation between serum KTR and both of the HOMA indexes. Serum KYNA concentrations correlated with KYN but not with TRP concentrations (Table 1).

## 4. Discussion

 The major findings of the present study are the correlations between serum concentration of KYN and scores of HOMA2-IR and HOMA-beta in HCV patients. As far as we are aware, this is the first observation of such a correlation. Considering that serum KYN concentrations used an index of IDO activity in HCV patients [9], our data suggested a possible involvement of upregulated of TRP-KYN metabolism in the development of IR in HCV patients.

We did not find correlation between IR indexes and KTR, a marker of IDO activity in clinical studies [13]. As it was indicated earlier (see Section 2), in HCV patients, serum KYN concentrations might be considered as an index of IDO activity [9]. 

Present finding of correlation between serum KYN and TRP with both HOMA-2-IR and HOMA-beta is in line with the reported induction of IR by surplus dietary TRP in pigs [19] and with recent observation of increased serum KYN in diabetes retinopathy patients [20]. 

Association between elevated KYN and TRP concentrations and IR might be a result or a cause of IR. It was considered that TRP-KYN metabolism might contribute to mechanisms of diabetes [11]. Recent review of clinical and experimental data suggested the involvement of KYN pathway of TRP metabolism in the development of IR since KYN and some of its derivatives affect biosynthesis, release, and activity of insulin [12]. 

**Figure 1:**
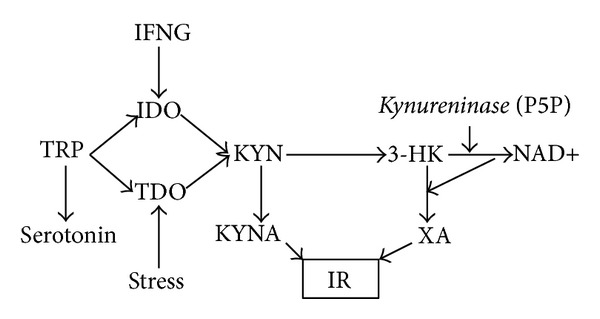
Kynurenic pathway of tryptophan metabolism and insulin resistance. Abbreviations: TRP: tryptophan; IFNG: interferon gamma; IDO: indoleamine 2,3-dioxygenase; TDO: TRP 2,3-dioxygenase; KYN: kynurenine; 3-HK: 3-hydroxyKYN; P5P: pyridoxal 5′-phosphate; NAD+: nicotinamide adenine dinucleotide; KYNA: kynurenic acid; XA: xanthurenic acid; IR: insulin resistance.

Diabetogenic effect of KYN and its derivatives, XA, 3-HK, and KYNA, (Figure 1) may be mediated by inhibition of pro-insulin synthesis in isolated rat pancreatic islets [21] and of insulin release from rat pancreas [22]. However, the effective concentrations (millimolar) of KYNA were much higher than its concentrations (micromolar) in pig's pancreatic juice [23]. The most plausible candidate for mediation of diabetogenic effect of upregulated TRY-KYN metabolism is XA. Increased urine excretion of XA was reported in type 2 diabetes patients in comparison with healthy subjects [24], while XA induced experimental diabetes in rats [25]. XA might contribute to the development of diabetes via formation of chelate complexes with insulin (XA-In) [26, 27] and induction of pathological apoptosis of pancreatic beta cells through caspase-3-dependent mechanism [28, 29]. Formation of XA from 3-HK depends on the vitamin B6 since its active metabolite, pyridoxal 5′-phosphate (P5P), is a cofactor of kynureninase, the enzyme, catalyzing 3-HK metabolism to NAD+ (Figure 1). P5P deficiency shifts 3-HK metabolism from formation of NAD+ to production of XA [30]. It is noteworthy that HCV infection is associated with significantly lowered P5P [31].

Evaluation of XA should be included in future studies of the role of TRP-KYN metabolism in mechanisms of HCV-associated IR. 

Mechanisms of IR in HCV might be different from those in non-HCV patients. Thus, we observed in agreement with previous finding that a positive correlation between HOMA-IR and HOMA-beta was reported in HCV patients [32], in comparison with negative correlation between HOAM-2IR and HOMA-beta in non-HCV patients [16]. In the present study, a strong positive correlation between HOAM-2IR and HOMA-beta was observed as well (Table 1).

## 5. Conclusions

 Our data of correlation between KYN and IR suggested the involvement of TRP-KYN metabolism in the development of IR in HCV patients. Detection and treatment of HCV-associated IR are of importance considering that HCV-associated IR may lead to resistance to antiviral therapy, hepatocarcinogenesis, and extrahepatic manifestations, including an increased risk of cardiovascular disorders [33]. TRP-KYN metabolism might be a new target for prevention and treatment of IR in HCV patients.

## Figures and Tables

**Table 1:** Kynurenines and insulin resistance in intent-to-treatment HCV patients.

*N* = 60	HOMA2-IR 1.3 (0.4–3.4)*	HOMA-beta 153 (57–395)*	KYN 1030 (480–3100)**	TRP 13550 (7000–27000)**	KTR 7.5 (3.2–14.2)*	KYNA 10 (4.3–36)
HOMA2-IR		*r* = 0.81 *P* < 0.0001	*r* = 0.32 *P* = 0.01	*r* = 0.31 *P* = 0.01	Not significant	Not significant
HOMA-beta	*r* = 0.81 *P* < 0.0001		*r* = 0.3 *P* = 0.02	*r* = 0.35 *P* = 0.01	Not significant	Not significant
KYN	*r* = 0.32 *P* = 0.01	*r* = 0.30 *P* = 0.02		*r* = 0.42 *P* < 0.0003	*r* = 0.62 *P* < 0.0001	*r* = 0.47 *P* = 0.0001
TRP	*r* = 0.31 *P* = 0.01	*r* = 0.35 *P* = 0.01	*r* = 0.42 *P* < 0.0003		*r* = −0.30 *P* = 0.005	Not significant
KTR	Not significant	Not significant	*r* = 0.62 *P* < 0.0001	*r* = −0.30 *P* = 0.005		Not significant
KYNA	Not significant	Not significant	*r* = 0.47 *P* = 0.0001	Not significant	Not significant	

*Median (50th percentile) (minimum-maximum).

**pmol/mL (50th percentile) (minimum-maximum).

## References

[1] Romero-Gómez M (2006). Insulin resistance and hepatitis C. *World Journal of Gastroenterology*.

[2] Moucari R, Asselah T, Cazals-Hatem D (2008). Insulin Resistance in chronic hepatitis C: association with genotypes 1 and 4, serum HCV RNA level, and liver fibrosis. *Gastroenterology*.

[3] Conjeevaram HS, Wahed AS, Afdhal N, Howell CD, Everhart JE, Hoofnagle JH (2011). Changes in insulin sensitivity and body weight during and after peginterferon and ribavirin therapy for hepatitis C. *Gastroenterology*.

[4] Huang JF, Yu ML, Dai CY (2013). Glucose abnormalities in hepatitis C virus infection. *Kaohsiung Journal of Medical Sciences*.

[5] Shlomai A, Mouler M, Rechtma E (2012). The metabolic regulator PGC-1a links hepatitis C virus infection to hepatic insulin resistance. *Journal of Hepatology*.

[6] Lecube A, Hernández C, Genescà J (2006). Proinflammatory cytokines, insulin resistance, and insulin secretion in chronic hepatitis C patients: a case-control study. *Diabetes Care*.

[7] Tanaka N, Nagaya T, Komatsu M (2008). Insulin resistance and hepatitis C virus: a case-control study of non-obese, non-alcoholic and non-steatotic hepatitis virus carriers with persistently normal serum aminotransferase. *Liver International*.

[8] Robinson CM, Hale PT, Carlin JM (2005). The role of IFN-*γ* and TNF-*α*-responsive regulatory elements in the synergistic induction of indoleamine dioxygenase. *Journal of Interferon and Cytokine Research*.

[9] Higashitani K, Kanto T, Kuroda S (2013). Association of enhanced activity of indoleamine 2, 3-dioxygenase in dendritic cells with the induction of regulatory T cells in chronic hepatitis C infection. *Journal of Gastroenterology*.

[10] Larrea E, Riezu-Boj JI, Gil-Guerrero L (2007). Upregulation of indoleamine 2,3-dioxygenase in hepatitis C virus infection. *Journal of Virology*.

[11] Connick JH, Stone TW (1985). The role of kynurenines in diabetes mellitus. *Medical Hypotheses*.

[12] Oxenkrug G (2013). Insulin resistance and dysregulation of tryptophan—kynurenine and kynurenine—nicotinamide adenine dinucleotide metabolic pathways. *Molecular Neurobiology*.

[13] Schroecksnadel K, Frick B, Winkler C, Fuchs D (2006). Crucial role of interferon-*γ* and stimulated macrophages in cardiovascular disease. *Current Vascular Pharmacology*.

[14] Cozzi A, Zignego AL, Carpendo R (2006). Low serum tryptophan levels, reduced macrophage IDO activity and high frequency of psychopathology in HCV patients. *Journal of Viral Hepatitis*.

[15] Turski WA, Nakamura M, Todd WP, Carpenter BK, Whetsell WO, Schwarcz R (1988). Identification and quantification of kynurenic acid in human brain tissue. *Brain Research*.

[16] Wallace TM, Levy JC, Matthews DR (2004). Use and abuse of HOMA modeling. *Diabetes Care*.

[17] Schwarcz R, Bruno JP, Muchowski PJ (2012). Kynurenines in the mammalian brain: when physiology meets pathology. *Nature Reviews Neuroscience*.

[18] Oxenkrug GF (2007). Genetic and hormonal regulation of tryptophan—kynurenine metabolism implications for vascular cognitive impairment, major depressive disorder, and aging. *Annals of the New York Academy of Sciences*.

[19] Koopmans SJ, Ruis M, Dekker R, Korte M (2009). Surplus dietary tryptophan inhibits stress hormone kinetics and induces insulin resistance in pigs. *Physiology and Behavior*.

[20] Munipally PK, Agraharm SG, Valavala VK, Gundae S, Turlapati NR (2011). Evaluation of indoleamine 2,3-dioxygenase expression and kynurenine pathway metabolites levels in serum samples of diabetic retinopathy patients. *Archives of Physiology and Biochemistry*.

[21] Noto Y, Okamoto H (1978). Inhibition by kynurenine metabolites of proinsulin synthesis in isolated pancreatic islets. *Acta Diabetologica Latina*.

[22] Rogers KS, Evangelista SJ (1985). 3-hydroxykynurenine, 3-hydroxyanthranilic acid, and o-aminophenol inhibit leucine-stimulated insulin release from rat pancreatic islets. *Proceedings of the Society for Experimental Biology and Medicine*.

[23] Kuc D, Zgrajka W, Parada-Turska J, Urbanik-Sypniewska T, Turski WA (2008). Micromolar concentration of kynurenic acid in rat small intestine. *Amino Acids*.

[24] Hattori M, Kotake Y, Kotake Y (1984). Studies on the urinary excretion of xanthurenic acid in diabetics. *Acta Vitaminologica et Enzymologica*.

[25] Kotake Y, Ueda T, Mori T (1975). Abnormal tryptophan metabolism and experimental diabetes by xanthurenic acid (XA). *Acta Vitaminologica et Enzymologica*.

[26] Ikeda S, Kotake Y (1986). Urinary excretion of xanthurenic acid and zinc in diabetes: (3) occurrence of xanthurenic acid-Zn2+ complex in urine of diabetic patients and of experimentally-diabetic rats. *Italian Journal of Biochemistry*.

[27] Meyramov G, Korchin V, Kocheryzkina N (1998). Diabetogenic activity of xanturenic acid determined by its chelating properties. *Transplantation Proceedings*.

[28] Malina HZ, Richter C, Mehl M, Hess OM (2001). Pathological apoptosis by xanthurenic acid, a tryptophan metabolite: activation of cell caspases but not cytoskeleton breakdown. *BMC Physiology*.

[29] Wang Q, Chen J, Wang Y (2012). Hepatitis C virus induced a novel apoptosis like death of pancreatic beta cells through a caspase 3-dependent pathway. *PLoS ONE*.

[30] Bender DA, Njagi ENM, Danielian PS (1990). Tryptophan metabolism in vitamin B6-deficient mice. *British Journal of Nutrition*.

[31] Lin CC, Yin MC (2009). Vitamins B depletion, lower iron status and decreased antioxidative defense in patients with chronic hepatitis C treated by pegylated interferon alfa and ribavirin. *Clinical Nutrition*.

[32] Helaly GF, Hussein NG, Refai W, Ibrahim M (2011). Relation of serum insulin-like growth factor-1 (IGF-1) levels with hepatitis C virus infection and insulin resistance. *Translational Research*.

[33] Trpkovic A, Stokic E, Radak D, Mousa S, Mikhailidis DP, Isenovic ER (2010). Chronic hepatitis C, insulin resistance and vascular disease. *Current Pharmaceutical Design*.

